# Short stay laparoscopic hysterectomy: An evaluation of feasibility and patient satisfaction

**DOI:** 10.52054/FVVO.13.4.039

**Published:** 2021-12-30

**Authors:** L Antoun, P Smith, Y Afifi, K Cullis, TJ Clark

**Affiliations:** Birmingham Women’s and Children’s NHS Foundation Trust, Birmingham, B15 2TG UK; Institute of Genomics and Cancer Sciences, University of Birmingham, B15 2TT UK; University of Birmingham, B15 2TT UK

**Keywords:** day case hysterectomy, enhanced recovery, laparoscopic hysterectomy, outpatient hysterectomy, short stay hysterectomy, 23 hour stay

## Abstract

**Background:**

Short-stay total laparoscopic hysterectomy (TLH) could lead to reduced hospital costs and decrease complications associated with hospitalisation such as hospital acquired-infection and venous thromboembolism.

**Objective:**

To evaluate the feasibility, safety and patient satisfaction of a novel short ‘less than 23-hour’ stay TLH protocol.

**Material and Methods:**

Prospective cohort study, at Birmingham Women’s Hospital, United Kingdom including eligible women undergoing TLH for benign indications or early stage cervical/endometrial cancer.

**Main outcome measures:**

Feasibility of discharge within 23-hours following TLH. Surgical complications and readmission rates were collected within 30-days of hysterectomy and patient’s satisfaction was assessed at 6-weeks.

**Results:**

Of the 128 eligible women, 104/128 women (81%) were discharged within 23-hours of admission, of which 62/104 or 60% (48.4% of the whole cohort) were discharged on the same day. Adenomyosis/fibroids, and previous caesarean sections were associated with a greater likelihood of stay beyond 23-hours (P<0.05). The overall complications rate was 13/128 (10%) with two grade-3 Clavien-Dindo intraoperative complications; one serosal bowel injury oversewn and one ureteric injury requiring reimplantation. The readmissions rate was 5/128 (4%). 94% of patients were ‘happy’ or ‘very happy’ with the pathway, although satisfaction was higher in short-stay patients (RR 1.2; 95% CI 0.95–1.94).

**Conclusion:**

Hospital discharge within 23-hours of TLH appears to be safe, feasible and acceptable to patients where a standardised, multidisciplinary care protocol is used.

**What is new?:**

Our study is the first prospective case series in the UK reporting the safety and acceptability for performing laparoscopic hysterectomy as a 23-hour day case procedure.

## Introduction

The proportion of laparoscopic hysterectomies in the United Kingdom, Europe and the United States of America have increased significantly compared to abdominal or vaginal approaches (Perino et al., 1999; Lonky et al., 2017; Madhvani et al., 2019; Meulen et al., 2018).

These endoscopic techniques combined with advances in technology and training have enabled shorter lengths of stay after gynaecological surgery with 42-97% success rate of ‘outpatient’ (‘day-case’ or ‘ambulatory’) laparoscopic hysterectomies performed in the US, and Europe (Moawad et al., 2017).The application of 23-hour discharge following laparoscopic hysterectomy in the UK is at present sporadic and infrequent, with no observational series published in the UK to date recording the safety and acceptability for performing laparoscopic hysterectomy as a 23-hour day case procedure. Although short-stay hysterectomy is associated with both clinical and economic benefits (Warren et al., 2009), the practice is not established. This may reflect a lack familiarity with early discharge.

In 2019, a guideline from the Association of Anaesthetists and the British Association of Day Surgery (Bailey et al., 2019) was published recommending a multidisciplinary approach between surgeons and the anaesthetic department, with agreed protocols for patient assessment to facilitate short postoperative stay. We designed a multidisciplinary enhanced recovery protocol and introduced a short stay hysterectomy pathway as part of a quality improvement project. The aim of our study was to evaluate if 23-hour discharge was feasible after total laparoscopic hysterectomy (TLH) in a unit unfamiliar with this concept, and to identify factors predictive for success and to evaluate patient satisfaction.

## Materials and methods

We performed a prospective, observational cohort study. All laparoscopic hysterectomies were undertaken at the Birmingham Women’s and Children’s Hospital (BWCH), a UK University Teaching Hospital. Data were prospectively collected over a two-year period from January 2019 and to December 2020 for women undergoing total laparoscopic hysterectomy (TLH) for benign or malignant disease.

### Pre-operative interventions

Patients were eligible for short-stay hysterectomy if they were between 18 – 60 years of age with an American Society of Anesthesiologists physical status score (ASA) I-II, a body mass index (BMI) of ≤ 35 and the surgical team did not anticipate any surgical complications. To meet the inclusion criteria, women needed to reside within one hours travel from the BWCH, and could arrange continuous home support for 24-hours. The criteria for exclusion were patients above the age of 60, a history of cardio-pulmonary compromise, renal disease, or sleep apnoea. Patients were provided with a diary that they were asked to complete in order to follow their journey, and provided with written information of what to expect after the procedure. The diary set up goals for recovery including: mobilising, diet, passing urine, satisfactory wound healing, pain control, and general health. Patients were made aware that completion of this diary was optional. Eligible patients were provided with a written information leaflet.

Women were advised to arrange appropriate post-operative support at home, and were instructed to eat up to 02.30 on the day of surgery and encouraged to drink water until two hours before surgery. All women were admitted on the day of surgery and given anti-embolic stockings. Low molecular weight heparin was used according to standard gynaecology venous thromboembolism (VTE) protocol based on risk assessment for VTE. All women were given a single dose of intravenous antibiotics immediately before the start of surgery.

### Intra-operative

Induction and maintenance anaesthetic regimens were according to the anaesthetist’s preference. Routine use of patient controlled anaesthesia (PCAs) was avoided and long acting narcotics were used sparingly to minimise somnolence and induction of paralytic ileus.

Intraoperatively local anaesthetic either at port sites and/or as laparoscopic transversus abdominis plane blocks (LTAPs) were used to allow early mobilisation. The technical steps for conducting a TLH were left to the discretion of the operating surgeon. The routine use of drains was avoided. Vaginal cuff was closed laparoscopically in all cases using continuous barbed suture.

### Post-operative

Nursing staff were provided with specific postoperative care protocols. Additionally, an escalation criterion to medical staff was provided. Women could drink normally and offered a light diet when hungry. Non-opioid, oral analgesics and routine anti-emetics were provided, and the urinary catheter was removed within 4-6 hours of the procedure. Patients could go home if they were able to void spontaneously and empty the bladder satisfactorily following catheter removal.

**Figure 1:**
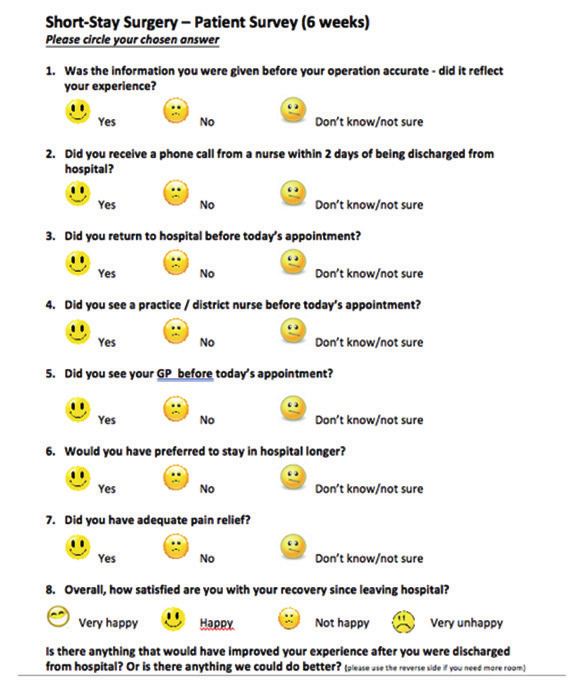
Patient’s survey at 6 weeks’ post-hysterectomy.

On discharge, nursing staff provided an emergency contact number for the hospital and a scheduled nurse-led follow-up phone call was arranged within 24-48 hours of discharge (Table I). Women were asked to complete a questionnaire at 6 weeks after the surgery (Figure 1) to assess the patient experience, compliance and satisfaction with the short-stay pathway.

**Table I:** Short stay hysterectomy pathway: Clinical discharge criteria and actions (nurse). The patient should NOT be discharged by a nurse if non-compliant with any of the criteria unless reviewed and sanctioned by a member of the medical staff.

Discharge criterion		Complied (tick box)	Actions if non-compliant
Uncomplicated surgery			
Surgery performed without complications (to be defined by surgeon)			-
EBL <500mL			-
Non-conversion to laparotomy			-
Post-operative observations ^1^			
Complied with MEWS chart – stable and normal vital signs and ability to maintain oxygen saturation levels >95% on room air			Escalate to medical staff
Post-operative progress ^1^			
Tolerated oral fluids and light diet without significant nausea / vomiting			-
Adequate control of nausea and vomiting			-
Adequate pain control with an oral regimen based upon paracetamol, NSAIDs and codeine but NO oral morphine required in the preceding 4 hours			-
Voiding spontaneously and emptying the bladder satisfactorily (bladder scan) OR willing to go home with an indwelling urinary catheter (removal within 48 hours)			-
Post-operative examination and tests			
Satisfactory abdominal examination = soft, minimally tender, no more than moderately distended (i.e. not tense) and unremarkable (dry and non-gaping) port site wounds			Escalate to medical staff
6 hour haemoglobin level is in the normal range AND has not dropped > 20g/L from the pre-operative level. ^3^			Escalate to medical staff
Medications to take home			
TTO’s prescribed by the medical staff and given to patient with instructions for use			
Routine analgesics - NSAIDs / paracetamol: Standard protocol: Ibuprofen 800mg tds x 4 days – BWCH pharmacy will issue 1 box of 24 x 400mg tablets (If contraindicated then alternative e.g. codeine based analgesic - codeine 60mg qds). Patients to be advised to revert to standard 400mg tds ibuprofen dose (buy from supermarket / pharmacy) after 4 days. Patients to be advised to take paracetamol as required (maximum 1g (2 tablets) 4 times per day).			
Routine stool softeners - lactulose / senna			
Routine antiemetics – cyclizine or ondansetron			
Exceptional (only if requested and prescribed by medical staff) – please tick all that apply:			Ignore and strike through if no exceptional TTOs prescribed
□	Opioids		
□	Antibiotics		
□	Hormone replacement therapy		
□	Low molecular weight heparin (clexane / dalteparin – according to standard gynaecology VTE protocol		
Post-discharge care			
Someone at home to act as a carer for the next 24 hours			
Patient phone number recorded to receive a post-operative phone call from the nursing staff the next day			
Written patient information and emergency contact numbers given			

^1^ Minimal 6-hours post-operative observation; ^2^ Escalate to medical staff if unsure or thought to be abnormal; ^3^ If abnormal medical staff may consider acceptable according to the clinical circumstances (e.g. patient well and: low starting haemoglobin concentration; <20g/L decrease; acute intra-operative bleed only).

### Theory/calculation

The primary outcome was feasibility of the short- stay TLH pathway. Secondary outcomes included the proportion of women achieving same day ‘outpatient’ discharge, rates of intra-operative and post-operative complications defined according to the Clavien-Dindo system (Dindo et al., 2004), representation, and readmission. In addition, data regarding patient’s satisfaction was collected at six weeks postoperatively.

### Statistical analysis

Descriptive analyses specifying the mean (m) for each quantitative variable and the percentages (%) and the size (n) are defined. Analyses were conducted using the graphpad statistics tool. A p = < 0.05 was considered significant. Women proactively converted to a laparotomy prior to commencing any surgical steps of a LH were excluded from analysis, but the numbers (%) presented.

## Results

### Baseline characteristics

**Figure 2:**
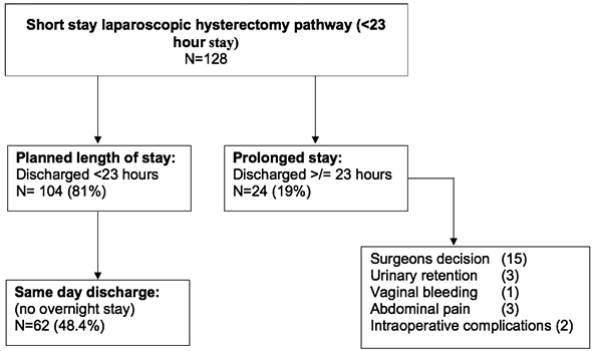
Flow chart for short-stay laparoscopie hysterectomy.

Two hundred-eighty-nine patient’s laparoscopic hysterectomies were performed of which, 128 (45%) women were placed upon the short-stay hysterectomy pathway and were included in the final analysis (Figure 2). Of the remaining 161 patients, 53/161 (33%) were not included due to social, medical or surgical ineligibility, and 108/161 patients (67%) were eligible, but were not enrolled due to a lack of familiarity with the newly implemented pathway. These 108 patients had a mean length of stay of 1.47, median of 1 (1-3) days. The average age and BMI of the study population were 47.4 years and 28 kg/m 2, respectively. The most common reasons for TLH amongst the 128 patients on the short-stay pathway were pelvic pain 58 (45%), followed by adenomyosis/fibroids 27 (21%), and heavy menstrual bleeding (HMB) 23 (18%). Patient’s clinical characteristics are summarised in (Table II). Only 58/128 (45%) of patients completed their diary with no significant difference between short-stay hysterectomy patients who ended up having <23hour stay compared to patients who had prolonged stay (38% compared to 79%; P = 0.4).

**Table II:** Clinical characteristics of the patients and predictors of length of stay.

All patients (n = 128)	Short-stay hysterectomy (<23hr) (n = 104)	>23hr stay (n = 24)	RR [95% CI]	P
Age </ = 60 years (Mean)	47.4 +/- 1.1	45.4 +/- 1.6	-	-
BMI (kg/m^2^) </=35 (Mean)	28 +/- 1.5	29.9 +/- 1.2	-	-
ASA I/II with no sleep apnoea	104 (100)	24 (100)	-	-
Caesarean history. n (%)	21 (20)	14 (58)	0.35[0.21-0.58]	<0.001
Laparotomy history. n (%)	2 (2)	3 (13)	0.15 [0.03-0.87]	0.03
Indications: n (%)				
Pelvic pain	46 (44)	8 (33)	1.32 [0.72-2.43]	0.4
Adenomyosis/Fibroids	17 (16)	10 (42)	0.39 [0.21-0.75]	0.004
Cervical pathologies ^a^	2 (2)	1 (4)	0.46 [0.04-4.88]	0.5
Endometrial cancer	9 (9)	3 (13)	0.69 [0.20-2.37]	0.6
Gender reassignment	4 (3.8)	0	2.14 [0.12-38.5]	0.6
BRCA risk reduction surgery	2 (2)	1 (4)	0.46 [0.04-4.89]	0.4
Menorrhagia	22 (21)	1 (4)	5.08 [0.72-35.8]	0.1
Dysmenorrhea (previous ablation)	2 (2)	0	1.19 [0.06-24.0]	0.1
Pre-existing operative findings: n (%)				
Endometriosis	19 (18)	6 (25)	0.73 [0.33-1.63]	0.4
Adhesions	13 (13)	7 (29)	0.43 [0.19-0.96]	0.04
Operating time: n (%)				
AM	68 (65)	18 (75)	0.9 [0.67-1.14]	0.3
PM	36 (35)	6 (25)		
Local infiltration/TAP block: n (%)	102 (98)	24 (100)	0.98 [0.95-1.01]	0.2
Pelvic drain	0	2 (8)	0.05 [0.002-0.96]	0.05
Intra-operative surgical complications				
Ureteric injury	0	1 (4)	0.08 [0.003-1.89]	0.1
Bowel injury (serosal)	0	1 (4)	0.08 [0.003-1.89]	0.1

### Clinical outcomes

104/128 of the eligible short-stay LH pathway patients (81%) were successfully discharged within 23-hours of their procedure and the majority were discharged on the same day (92/128; 72%). The mean length of stay was 0.77, median of 1 (0-5) days. The commonest reason for stay beyond 23-hours was a proactive decision by the surgical team for prolonged observation due to a more complex procedure than anticipated 15/24 (63%). Other reasons were urinary retention 3/128 (2.3%), vaginal bleeding 1/128 (4%), abdominal pain 3/128 (12.5%), and intra-operative complications 2/128 (8%). Intra-operative complications included one ureteric injury (0.8%), followed by stenting and one serosal bowel injury (0.8%), which was repaired using p-dioxanone (PDS 3-0). Both injuries were identified and managed intra-operatively.

Compared with patients who were successfully compliant with the short-stay pathway, those who had prolonged stay were more likely to have previous laparotomy (3, 13% vs. 2, 2%, P= 0.03), previous caesarean section (14, 58% vs. 21, 20%, P= <0.001), and adenomyosis/fibroids (10, 42% vs. 17, 16%, P = 0.004).

The timing of surgery on an all-day operating list did not influence the likelihood of being discharged within 23-hours of the procedure. 68/86 (79%) of women who had their surgery in the morning were compliant with the pathway compared to 36/42 (86%) who had their surgery in the afternoon; [relative risk (RR)= 0.9 (95% CI 0.78-1.08; P= 0.3)]. Almost all patients had either LTAP block, or local anaesthetic infiltration to the trocar sites, without any significant difference between those women discharged within 23-hours and those who had prolonged stay (102/104, 98% vs 24/24,100%, P= 0.2). However, patients who had a pelvic drain inserted were more likely to have prolonged stay 2/24 (8%) [relative risk= 0.05 (95% CI 0.002-0.96; P= 0.05)].

The readmission rate following discharge was 5/128 (3.9%) at 30 days’ post hysterectomy (Table III). Three of these readmissions were within seven days of surgery. Of the five readmissions, three were due to Clavien-Dindo grade 1 post- operative complications; one upper respiratory tract infection, one pyelonephritis, and one port- site wound dehiscence. The other patient was re- admitted on day two postoperatively and had a pelvic collection requiring laparoscopy and drainage in theatre (Clavien-Dindo grade 3b). One patient was readmitted 10 days post-operatively with a ureteric-vaginal fistula following an unrecognised thermal ureteric injury (Clavien-Dindo grade 3b). There were no relevant readmissions identified after 30 days. In total two patients required a return to theatre (2/128; 1.5%).

**Table III:** Post-operative complications.

All patients (n = 128)	Short-stay hysterectomy (<23hr) (n = 104)	>23hr stay (n = 24)	RR [95% CI]	P
Clavien-Dindo grade 1 post-op complications n (%):	4 (3.8)	5 (21)	0.18 [0.05-0.64]	0.008
Pyelonephritis (day 4)	0	1 (4)	0.08 [0.00-1.89]	0.1
Infection (day 2-7)	2 (2)	1 (4)	0.46[0.04-4.88]	0.5
Abdominal pain (day 1-5)	0	3 (13)	0.03[0.00-0.64]	0.02
Wound dehiscence (day 2)	1 (2)	0	0.71 [0.03-17.0]	0.8
Clavien-Dindo grade 3 post-op complications requiring return to theatre n (%):	2 (2)	2 (8)	0.23 [0.03-1.56]	0.1
Vaginal bleeding (day 0)	0	1 (4)	0.08 [0.00-1.89]	0.1
Pelvic collection (day 2)	1 (1)	0	0.71 [0.03-17.01]	0.8

### Patient’s satisfaction (Table IV)

**Table IV:** Patient experience: Postal questionnaire at 6 weeks post laparoscopic hysterectomy.

Questionnaire (6-12 weeks following discharge) n: 62/128 (%):	Short-stay hysterectomy (<23hr) (n=45/104)	>23hr stay (n=17/24)	RR [95% CI]	P
Accurate pre-op information n (%):				
Yes	42 (93)	12 (71)	1.3 [0.96-1.81]	0.08
No	2 (4)	3 (18)		
Not sure	1 (2)	2 (12)		
Nurse phone within 2 days from discharge n (%):				
Yes	17 (38)	1 (6)	6.4 [0.92-44.6]	0.06
No	23 (51)	16 (94)		
Not sure	5 (11)	0		
Return to hospital within 6 weeks from discharge n (%):				
Yes	5 (11)	2 (12)	0.4 [0.02-5.70]	0.9
No	38 (84)	15 (88)		
Not sure	2 (4)	0		
GP review within 6 weeks from discharge n (%):				
Yes	1 (0.1)	1 (6)	0.4 [0.02-5.70]	0.5
No	43 (96)	16 (94)		
Not sure	1 (2)	0		
Prefer prolonged hospital stay n (%):				
Yes	6 (13)	8 (47)	0.3 [1.12-0.74]	0.009
No	38 (84)	7 (41)	2.2 [1.20-3.93]	0.01
Not sure	1 (2)	3 (18)		
Adequate pain relief n (%):				
Yes	39 (87)	12 (71)	1.2 [0.88-1.70]	0.2
No	6 (13)	5 (29)		
Not sure	0	0		
Satisfied with short-stay hysterectomy n (%):				
Very Happy	34 (76)	6 (35)	0.5 [0.2-0.9]	0.02
Happy	10 (22)	8 (47)	2.1 [1.0-4.5]	0.05
Not happy	1 (2)	5 (29)		

The questionnaire response rate at six weeks following surgery was 62/128 (48%). Overall, most women were satisfied with their experience of short-stay hysterectomy (56/62; 90%). Assuming all non-responders were either dissatisfied or satisfied, the proportion of patients being satisfied with their experience of short-stay hysterectomy was between 56/128 (48%) and 122/128 (95%).

Significantly more women who were successfully discharged within 23 hours 44/45 (98%) were ‘happy’ or ‘very happy’ with their experience than those women 12/17 (71%) requiring prolongation of hospital stay; [RR= 1.3 (95% CI 1.02-1.89; P= 0.03)].

Significantly more women in the 23-hour stay group felt that early discharge was appropriate (38/45 [84%]), compared to those women in the prolonged stay group (7/18 [41%]; RR= 2.2; 95% CI 1.20 – 3.92, P= 0.01). Most women in each group felt that pain relief was adequate [(39/45 [87%] vs 12/17 (71%); RR= 1.2 (95% CI 0.88 – 1.70; P=0.2)]. Only 18/128 (14%) of women received a documented nurse call within 24-48 hours of discharge, and 2/62 (3%) had to see their general practitioner within six weeks of discharge for pain relief prescription.

## Discussion

### Main findings

We have presented our novel protocol for short- stay hysterectomy and the initial clinical outcomes following its implementation. This ‘less than 23- hour stay’ LH care pathway for benign conditions, and early-stage cancer patients being discharged within 23 hours of admission, with rates of complications and representations/readmissions comparable to conventional care and high rates of patient satisfaction (Meulen et al., 2018; Jennings et al., 2015; Bruneau et al., 2015). Moreover, 48% of all patients, accounting for 60% of all those discharged within 23-hours, went home on the same day as surgery, avoiding overnight stay. Almost one in five women (19%) required stay beyond the planned 23-hours following TLH. The main barrier to compliance was the decision of the surgical team for prolonged observation due to unanticipated surgical complexity. As the care pathway becomes firmly embedded, confidence may increase and prolonged admissions reduced.

Patients with fibroids/adenomyosis were more likely to have prolonged stay beyond 23-hours compared to patients who had a hysterectomy for other indications. Patients with a prior caesarean section, known adhesions or intra-operative pelvic drain insertion were also more likely to stay beyond 23-hours. The enhanced surgical challenges presented by larger uteri associated with adenomyosis and fibroids, and adhesions resulting in longer duration of surgery may possibly explain the relatively prolonged post-operative recovery. Placement of a pelvic drain being indicative of complex surgery and concerns over post-operative bleeding.

### Strengths and limitations

Data were collected prospectively from 128 consecutive women undergoing LH who had been placed on our pathway. However, scrutiny of our electronic operating theatre coding system showed that 289 laparoscopic hysterectomies were undertaken during our study period. Retrospective comparison of the 161 patients not included, revealed that 108 patients were eligible but not included. However, these patients were not selectively excluded but rather were omitted because of a lack of familiarity with the pilot care pathway. Based upon these data, by applying the inclusion criteria presented in our protocol, we estimate that over 80% of women undergoing LH will be eligible for this short-stay care pathway.

The low response rate of around 50% to our 6-week patient satisfaction questionnaire limits robust clinical inferences being drawn. However, results were generally more favourable in women discharged within 23-hours.

### Comparison with the existing literature

Concerns about early discharge after hysterectomy include high readmission rates and missed complications. In our study the readmission rate was low and reasons in this cohort included chest infection, urinary infection, ureterovaginal fistula and pelvic collection. It is unlikely that keeping these patients in for one or two more days would have been long enough for these complications to manifest clinically. Rather patient education about concerning symptoms along with 24/7 contact numbers to allow clinical assessment can identify problems early and improve outcomes.

In England, the mean rate of emergency readmission within 30 days of laparoscopic hysterectomies is 4.9% (Meulen et al., 2018). Our findings are in keeping with previous observational series from the USA and Europe examining the feasibility of same-day TLH reported success rate of 41.7% to 97% with readmission rate ranging from 1.4% to 8%, and up to 6% return to theatre in the first 30 days following the procedure (Doliyet et al., 2020; Thiel and Gamelin, 2003; Rivard et al., 2015; Summitt et al., 1992; Alperin et al., 2012). Furthermore, there was 20% of patients requiring prolonged hospitalisation (Doliyet et al., 2020), and 3.4% rate of conversion to laparotomy (Alperin et al., 2012). Data from a recent, large, multicentre RCT in the UK (Cooper et al., 2019) comparing laparoscopic supracervical hysterectomy and endometrial ablation, showed that post- operative hospital stay was less than 24 hours in 68% of women who were randomly allocated to laparoscopic supracervical hysterectomy, this may reflect the lack of a structured model as set out and assessed in our study.

In the context of same-day discharge following TLH, a recent RCT demonstrated that voiding within 6 hours after surgery was associated with a successful same-day discharge (Sandberg et al., 2019). The main reasons given for prolonged stay in other observational studies were pain, nausea and vomiting (Dindo et al., 2004; Jennings et al., 2015; Bruneau et al., 2015; Dolivet et al., 2020). In our study, less than 3% of women had prolonged stay due to urinary retention or excessive pain, with nausea and vomiting not recorded as a prime reason for protracted admission. This may reflect our intraoperative and postoperative protocol, removing the catheter within 4-6 hours after surgery and offering patients regular non-opioids, and antiemetic medication.

### Implications for clinical practice and research

In 2020, The British Association of Day Surgery (BADS) in collaboration with Getting It Right First Time (GIRFT., 2020) produced a Day Surgery Delivery Pack highlighting the importance of considering the option of day surgery wherever possible, especially at a time when the NHS needs to re-start and catch up with demand for elective surgery following the Covid-19 pandemic.

Day surgery is beneficial for both patients and the hospital as it helps to reduce waiting times. It also has financial implications by releasing valuable bed stock, reducing the burden of highly skilled staff during out-of-hours cover and minimising the risks of hospital acquired infection and venous thromboembolism. This study shows that short-stay hysterectomy is feasible and safe without increased rates of readmission, complications or reduced patient satisfaction. Moreover, this novel pathway can be successfully introduced into hospitals where traditional practices regarding post-operative length of stay are embedded.

Patients should be counselled that most can be discharged on the same day as their surgery without the need for hospital admission. However, there is a 1 in 5 chance of needing hospital stay beyond 23-hours and the likelihood of needing longer stay is higher in the presence of adenomyosis, fibroids, previous caesarean sections and adhesions. They should also be aware that rates of satisfaction are high and be reassured that the need for readmission or of suffering complications are no higher than conventional care protocols. Large, multi-centre, randomised controlled trials comparing day- case, less than 23 hour stay and conventional care protocols are needed to evaluate the magnitude of any differences in rates of complications such as hospital acquired infections, rates of venous thromboembolism and patient satisfaction. Collection of prospective observational, real world data (e.g. establishing patient registries) is needed to better assess the safety of same day and short stay hysterectomy.

## Conclusion

We developed and introduced a novel short-stay ‘less than 23-hour stay’ laparoscopic hysterectomy pathway. Such a pathway for TLH is feasible, with most patients being discharged on the day of surgery, and associated with high levels of patient satisfaction.
